# Ultrasonic Synthesis of Magnesium–Iron Layered Double Hydroxides and Their Sorption Properties Toward Chromate Anions

**DOI:** 10.3390/ijms27104251

**Published:** 2026-05-10

**Authors:** Roman A. Golubev, Omar M. Khubiev, Daria I. Semenkova, Linh V. Nguyen, Anton R. Egorov, Nikolai N. Lobanov, Rovshan H. Nazarov, Victor N. Khrustalev, Anatoly A. Kirichuk, Vasili V. Rubanik, Alexander G. Tskhovrebov, Andreii S. Kritchenkov

**Affiliations:** 1 Department of Human Ecology and Bioelementology & Department of General and Inorganic Chemistry, RUDN University, 6 Miklukho-Maklaya St., Moscow 117198, Russia; asdfdss.asdasf@yandex.ru (R.A.G.); ihubievomar1@gmail.com (O.M.K.); darya.semenkova02@mail.ru (D.I.S.); linhno2109@gmail.com (L.V.N.); sab.icex@mail.ru (A.R.E.); nnlobanov@mail.ru (N.N.L.); khrustalev-vn@rudn.ru (V.N.K.); kirichuk-aa@rudn.ru (A.A.K.); alexander.tskhovrebov@gmail.com (A.G.T.); 2 Institute of Technical Acoustics NAS of Belarus, Ludnikova Prosp. 13, 210009 Vitebsk, Belarus; v.v.rubanik@tut.by; 3 Institute of Chemistry of Additives, Beyukshor Highway, Block 2062, Baku 1029, AZ, Azerbaijan; rovsenhafiz@mail.ru

**Keywords:** layered double hydroxides, ultrasound, chromate sorption, X-Ray diffraction analysis, sonochemistry

## Abstract

Layered double hydroxides (LDHs) are promising anion sorbents, but conventional Mg–Fe LDH synthesis requires prolonged aging. The effects of ultrasound application stage on Mg–Fe LDH microstructure and chromate uptake remain insufficiently clarified. This study compared ultrasonic treatment during and after coprecipitation and related XRD-derived microstructural descriptors to Cr(VI) sorption. Mg–Fe LDHs were synthesized using 28 or 40 kHz ultrasound during or after coprecipitation and 1.7 MHz ultrasound after coprecipitation; 24 h thermal aging was used as a reference. The products were characterized by ICP-MS, FTIR, TGA/DSC, SEM, and XRD and tested for chromate adsorption, kinetics, recyclability, multicomponent-solution performance, and soil Cr(VI) immobilization. Fifteen minutes of ultrasonication yielded Mg/Fe ≈ 2 LDHs and shortened synthesis compared with 24 h aging. Ultrasound during coprecipitation at 28 kHz gave the best sorbent, increasing experimental adsorption capacity to 80.35 mg/g versus 53.70 mg/g for the reference LDH. Sorption followed pseudo-second-order kinetics and was best described by the Freundlich model. In a multicomponent solution, this sample removed 68% Cr(VI) at 1.0 g/L and reduced water-soluble Cr(VI) in soil from 14.31 to 0.26 mg. Ultrasound application during coprecipitation improves Mg–Fe LDH structure-related characteristics and chromate sorption.

## 1. Introduction

Layered double hydroxides (LDHs) are inorganic materials constructed from positively charged layers of di- and trivalent metal cations coordinated to hydroxide groups and anions [1,2]. The interlayer space of LDHs contains anions and water molecules, which can be readily replaced by various other anionic molecules, and this characteristic property of LDHs provides them with a rich intercalation chemistry [3,4]. Not surprisingly, LDHs have been successfully studied and used as sorbents for a wide variety of organic and inorganic compounds (heavy metals, dyes, antibiotics, herbicides and pesticides, etc.) [5,6]. LDHs are also of interest as controlled drug release systems, catalysts, nanoreactors, and carriers of various nanosystems, such as quantum dots [5,7,8,9,10,11]. However, these potential applications of LDHs are also driven by their outstanding sorption properties [12]. Among different LDHs of various M^2+^–M^3+^ compositions, Mg–Fe LDHs are widely regarded as one of the most biocompatible LDH systems due to the use of physiologically relevant and environmentally friendly cations (Mg^2+^ and Fe^3+^), which exhibit lower toxicity compared to commonly used alternatives, such as Al^3+^- or Co^2+^-containing LDHs [13,14,15,16,17,18,19].

Conventional synthesis of LDH involves the coprecipitation method followed by prolonged (24 h or more) heating of the reaction mixture. However, over the past two decades, a growing number of publications have focused on ultrasonic treatment of the reaction mixture, which allows for avoiding prolonged heating and significantly reducing the synthesis time. In principle, ultrasound can be applied during or after coprecipitation, which is addressed in a recent review that focuses on the ultrasonic synthesis of LDH [20]. Currently, Scopus returns approximately 240 papers for the search query “layered double hydroxides ultrasound,” but only 13 of them (just over 5%) focus on magnesium–iron LDHs. Available papers, in one form or another, combining Mg-Fe LDH and ultrasound are summarized in Table 1.

Of these 13 articles (Table 1), entries 5, 6, 9, and 11 suggest the use of ultrasound during the application of LDHs, while the remaining nine papers describe the use of ultrasonic vibrations for the synthesis of magnesium–iron LDHs or their composites. Table 1 clearly shows that there are no preparative studies in the literature that compare two different ultrasonic modes (during or after coprecipitation) under the same conditions.

In studies focusing on the ultrasonic synthesis of magnesium–iron LDHs, the authors primarily discuss the effect of ultrasound on the degree of crystallinity. They use a limited set of models to describe the structural features of the prepared LDHs. We were unable to find any research papers that compare the application of ultrasound during and after coprecipitation and discuss the influence of these synthetic ultrasound modes on the finer structural features of magnesium–iron LDHs and their sorption properties. We aimed to systematically compare and quantify the relationships between ultrasonic synthesis modes and the resulting structural properties of Mg–Fe LDHs. We also strove to evaluate the sorption properties of the obtained LDHs toward an environmental pollutant (hexavalent chromium, i.e., chromate anions) and identify the relationships between the synthetic mode of applied ultrasound, the structural features of the synthesized LDHs, and their sorption capacity.

Thus, we hypothesized that the stage of ultrasonic application (during vs. after coprecipitation) systematically affects the resulting crystallite size and microstrain of Mg–Fe LDHs, which in turn correlates with their sorption performance. While this hypothesis is not mechanistic in nature, it provides an experimentally testable structure–property relationship that can serve as a basis for future mechanistic investigations. Although the present study does not resolve the mechanistic aspects of acoustic cavitation, the observed trends are consistent with its influence on nucleation and particle aggregation processes (and this is undoubtedly of interest for future mechanistic studies).

A detailed description of the study and its results is discussed in the sections that follow.

## 2. Results and Discussion

### 2.1. Preparation of LDH

We used a coprecipitation method to prepare LDH with a magnesium to iron ratio of 2:1. Dropwise addition of alkali to a solution of magnesium and iron nitrates leads to the following chemical reaction:

2Mg^2+^ + Fe^3+^ + 6OH^−^ + NO_3_^−^ + *n*H_2_O = [Mg_2_Fe(OH)_6_](NO_3_) × *n*H_2_O.

During the coprecipitation stage, we added the alkali solution to the metal salt solutions for the same amount of time (15 min). For traditional synthesis, after coprecipitation, we maintained the reaction mixture under prolonged heating (24 h at 70 °C). Ultrasonic synthesis was performed in four different modes, which involved varying (i) the frequency (28 or 40 kHz) and (ii) the order of ultrasound application (after or during coprecipitation). The code names of the resulting samples and the key synthesis conditions are summarized in Table 2.

**Figure 1:**
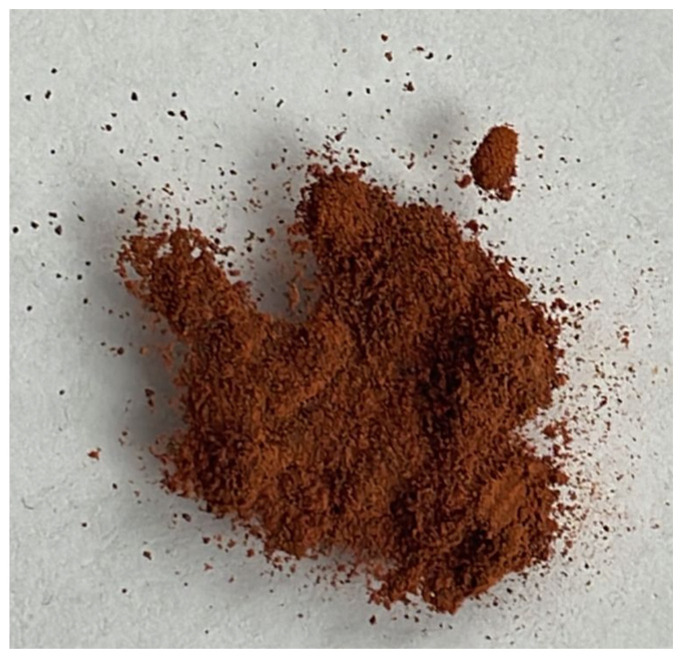
Typical appearance of the obtained LDHs in example 28.2.

The LDHs were obtained as brown powders (Figure 1) and characterized by a set of physicochemical analytical methods.

The ratio of magnesium and iron, as well as nitrate ions in all obtained samples, corresponds to the simplified formula [Mg_2_Fe(OH)_6_](NO_3_) × 2H_2_O, which was confirmed by inductively coupled plasma mass spectrometry (for Mg and Fe) and titrimetric analysis for nitrate ions. The refined values of the Mg/Al ratio are presented in Table 2. The water content was calculated from thermogravimetric analysis (water evaporation accounts for 11–12% of the mass loss).

### 2.2. FTIR Characterization of LDH

**Figure 2:**
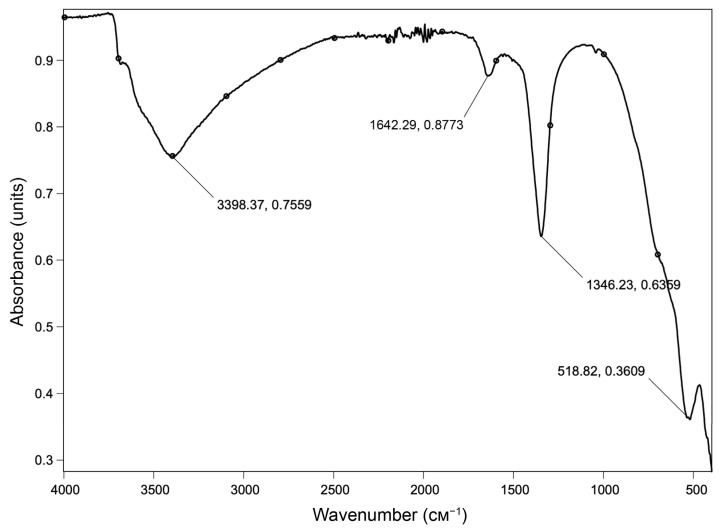
Typical FTIR spectrum of the prepared LDHs in example 28.2.

The IR spectra of LDHs are completely uniform and contain characteristic bands typical for this class of materials (Figure 2, in example 28.2). A pronounced absorption band at 3398 cm^−1^ is attributed to the O–H group stretching vibrations (which are characteristic of hydroxide structural motifs and water molecules). The bending vibrations of water molecules result in a broad, low-intensity absorption band at 1642 cm^−1^. Nitrate anion N–O bond vibrations manifest as a narrow, high-intensity absorption band at 1346 cm^−1^ and a broad, low-intensity band at 750 cm^−1^. A medium-intensity absorption band with a sharp shoulder at 519 cm^−1^ indicates the presence of metal–oxygen coordination bonds in example 28.2. Characteristic groups and corresponding wave numbers in IR spectra of other LDHs (T, 28.1, 40.1, 40.2, and 1.7) are summarized in Table 3.

### 2.3. TGA/DSC Characterization of LDH

**Figure 3:**
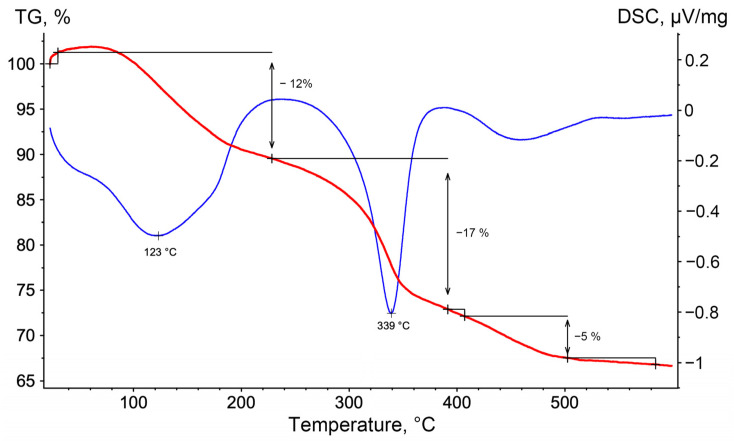
TGA(red)/DSC(blue) curves of the prepared LDHs in example 28.2.

The thermal decomposition patterns of the synthesized LDHs are also almost identical. Thermal degradation progresses in three stages, as seen in Figure 3 (in example 28.2). The first stage, primary dehydration, involves removal of both physically adsorbed and interlayer water, resulting in about 12% weight loss. The second stage, secondary dehydration or dehydroxylation of hydrotalcite-like layers, transforms the hydroxide layers into the oxide layers, with an approximate weight loss of 17%. The third stage involves denitrification of the interlayer space, with about 7% mass loss. All stages are endothermic processes, as shown by the blue curve (Figure 3). Notable endothermic effects are seen in the first two stages at 123 °C and 339 °C, respectively, whereas the third stage’s endothermic effect is marginal. The key parameters of the thermal decomposition stages of other LDHs (T, 28.1, 40.1, 40.2, and 1.7) are summarized in Table 4.

### 2.4. SEM Characterization of LDH

**Figure 4:**
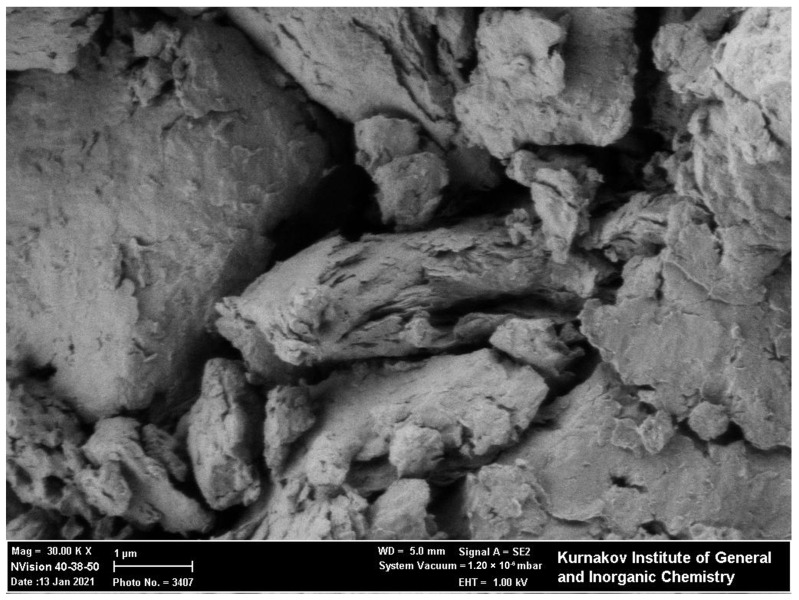
SEM image of LDHs in example 28.2.

The SEM image of sample 28.2 (Figure 4) shows aggregates of irregular particles containing some plate- or flake-like fragments, which is generally consistent with the layered nature of LDH materials. However, the observed morphology should not be interpreted as a uniformly platelet or predominantly flake-like morphology. In the present study, SEM characterization was used only as a representative qualitative visualization of the material morphology and was not used to establish correlations between synthesis conditions, structural parameters, and sorption properties. A systematic SEM comparison of all synthesized samples, including lower-magnification images, would be useful for a more complete morphological analysis but was beyond the scope of the present work.

### 2.5. Powder X-Ray Diffraction Characterization of LDH

**Figure 5:**
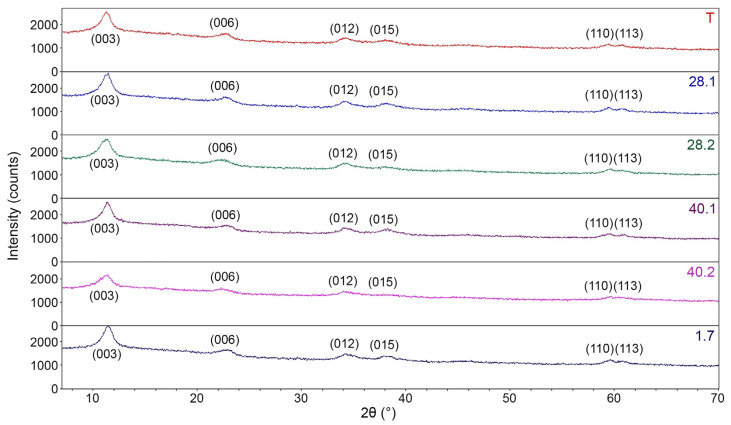
Diffraction patterns of the obtained LDH.

**Figure 6:**
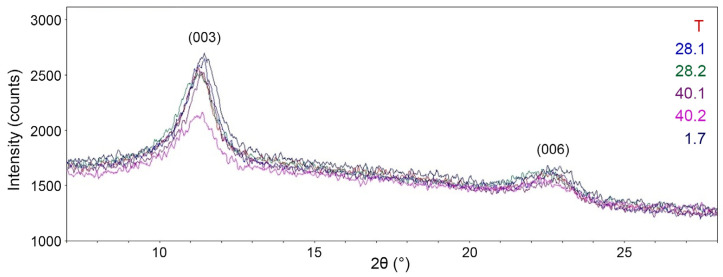
Enlarged fragment of superimposed diffraction patterns (reflection peaks 003 and 006).

Figure 5 exhibits the complete diffraction patterns of the synthesized LDH, while Figure 6 compares the (003) and (006) reflection peaks. All diffraction patterns (Figure 5) are typical for LDH (IDD PDF-2 № 14-0191, № 24-1091, № 53-1185, etc.). Thus, all diffraction patterns display pronounced reflection peaks at ~11.3° and 22.7° 2θ, which correspond to the (003) and (006) planes. The diffraction patterns also reveal broad asymmetric peaks at higher 2θ values of ~34.3°, 38.4°, etc., which are typical of LDH. Figure 6 shows that the intensity of the (003) and (006) reflection peaks for LDH 40.2 is significantly lower compared to the similar peak intensities of other samples. This directly indicates a significantly worse crystallinity of 40.2 compared to other synthesized LDH species. For all samples, the interlayer distance d_003_ is 0.77–0.78 nm and is in good agreement with the data from IDD PDF-2 No. 24–1091 and others.

We also calculated the crystal lattice parameters in the rhombohedral syngony system (space group R-3m) using five lines, which are the most distinct and characteristic of this class of materials. Thus, we obtained the following values for the prepared LDH: a = 3.100–3.104 Å, c = 23.5–23.7 Å, and V = 196–197 Å^3^.

We performed a full-profile analysis to study the microstructure of the obtained LDHs using X-ray diffraction data. We used single profiles of the (003) and (006) reflection peaks for our analysis, as they are the most statistically significant and allow us to evaluate the microstructural features of LDHs and the degree of order in their packing along the c-axis.

The integral broadening of peaks in diffraction patterns can be caused by two factors. First, the integral broadening is affected by the degree of structural perfection. The greater the integral broadening, the less perfect the crystal structure of the sample. Second, the integral broadening can be caused by the size of the micro- (nano-) blocks, since the size of the coherent X-ray scattering regions (D) corresponds to the grain size of a polycrystal or the size of domains in a textured single crystal. A decrease in the particle block size and/or an increase in microstrain leads to peak broadening in the diffraction pattern. The broadening due to the small size of the coherent scattering regions does not depend on the order of reflection and can be calculated using the Debye–Scherrer formula [31]:

$$\beta_{D}=\frac{K\lambda }{D\cos\theta },$$

where K is the shape factor (can take different values, most often K ≈ 0.9–1.0), λ is the radiation wavelength, D is the size of the microparticles, θ is the Bragg angle, and β_D_ is the size broadening.

The broadening caused by microdeformations depends on the order of reflection and can be calculated using the Stokes and Wilson formula [32]:

$$\beta_{s}=4\varepsilon \times tg\theta ,$$

where β_S_ is the strain broadening and ε = <∆d/d> is the relative deformation of the crystal lattice.

However, the experimental characteristics of the reflection peak profiles are related not only to these sample properties but also include so-called instrumental broadening. Instrumental broadening is determined by the individual hardware characteristics of the diffractometer, including its geometry (beam divergence, collimator slit width, etc.), the spectral purity of the radiation, the detector resolution, etc. Neglecting this effect leads to a systematic underestimation of the calculated crystallite sizes and an increase in microstrains, since the Scherrer, Stokes, and Wilson formulas interpret the total broadening solely as due to microparticles or microstrains.

The physical broadening effect is not a simple superposition but rather a convolution of the functions of the aforementioned effects. Depending on the function types used to approximate the peak profiles, we will use the following equation to calculate the physical broadening FW(S). This equation only takes into account the presence of very small particles or microscopic deformations and is described by the following mathematical formula:

FW(S)^N^ = FW^N^ − FW(I)^N^,

where N is the degree (takes a value from 1 to 2 depending on the peak describing function (Cauchy function, Gaussian function, or their combination), FW(S) is the broadening caused by microdeformations or small particle size, FW(I) is the instrumental broadening, and FW is the total peak broadening.

To estimate the instrumental broadening of diffraction reflections, we determined the instrumental broadening function (IBF) of the Tongda TD-3700 diffractometer, which was used to record diffraction patterns in the current study. To determine the IBF, we recorded the X-ray diffraction spectrum of a polycrystalline annealed Si sample in the 2θ range from 30° to 140°. Using full-profile analysis, we then obtained the IBF, which we used for further calculations.

Below is the calculation of the microstructure parameters based on the profile analysis of single peaks of reflections (003) and (006) in the approximation that the effect is associated either with the sizes of microparticles or with their microdeformations. Table 5 shows the obtained values of the crystallite sizes according to the Scherrer equation taking into account the instrumental function, calculated in three approximations: using the full width at half maximum of the peak (FWHM) − FW(S) = β = FWHM with powers N = 1 and N = 2 and the integral broadening (breadth) − FW(S) = β = breadth of single peaks of reflections (003) and (006) (D_003_ and D_006_), as well as their arithmetic mean values D_m_ and regression mean values (taking into account errors) D_w_ for the synthesized LDH species T, 28.1, 28.2, 40.1, 40.2, and 1.7.

The crystallite size values calculated using various models, presented in Table 5, differ slightly between the models; however, all correlations remain. The crystallite sizes of samples T, 28.1, 40.1, and 1.7 are quite large and similar in magnitude. We also observed similar, but smaller, crystallite sizes for samples 28.2 and 40.2. However, sample 40.2 was crystallized significantly worse than the other samples, and this should be kept in mind when comparing its sorption properties with those of the other samples. Thus, among samples T, 28.1, 28.2, 40.1, and 1.7, sample 28.2 exhibits the smallest crystallite sizes. It should be noted that the crystallite sizes determined from X-ray diffraction correspond to the sizes of coherent scattering domains and do not necessarily reflect the size of aggregated particles or the accessible surface area of LDH materials. In general, based on all three calculation models given in Table 5, we observe the following series of samples in order of decreasing crystallite sizes: T-40.1-1.7-28.1-28.2-40.2.

Table 6 contains data on the regression average microstrains, which we calculated using the Stokes and Wilson formula taking into account the instrumental function in three approximations: using the full width at half maximum of the peak (FWHM) FW(S) = β = FWHM with powers N = 1 and N = 2 and the integral broadening (breadth) FW(S) = β = breadth of single peaks of reflections (003) and (006) for all samples (T, 28.1, 28.2, 40.1, 40.2 and 1.7). The highest values of the average microstrains ε are characteristic of samples 28.2 and 40.2. Among the other samples, microstrains increase in the series T-1.7-40.1-28.1. An increase in microstrain may indicate a higher degree of lattice distortion or structural defects; however, the specific nature and distribution of these defects are not resolved within the present study.

If the sample contains microdeformations and consists of very small particles, it is customary to use the Williamson–Hall method [33], which is the result of a combination of the Scherrer and Stokes equations:

$$\beta =\beta_{D}+\beta_{s}=\frac{K\lambda }{D\cos\theta }+4\eta \times tg\theta ,$$

The graph of this equation allows us to calculate the values of D and ε.

We also calculated the microstructure parameters using the full-profile analysis using the profiles of the reflection peaks (003) and (006) of the (00h) plane series as the most statistically significant. This allowed us to consider the simultaneous influence of both the sizes of microparticles and their microdeformations. Table 7 presents the microstructure characteristics of samples T, 28.1, 28.2, 40.1, and 1.7 obtained by the Williamson–Hall method, taking into account the instrumental function and calculated in three approximations: using the full width at half maximum (FWHM) − FW(S) = β = FWHM with powers N = 1 and N = 2 and the integral broadening (breadth) − FW(S) = β = breadth of single reflection peaks (003) and (006).

The calculated values of crystallite size D_eff_ and microstrain ε indicate the presence of very fine particles and their deformation. The previously obtained correlations were preserved. Samples T, 40.1, and 1.7 are the closest in both size and microstrain (a slight increase in microstrain is observed in the series T-1.7-40.1). Sample 28.2 is characterized by the smallest particle sizes and, at the same time, the greatest microstrain. The microstructural parameters of sample 28.1 are between the characteristics of samples T, 1.7, and 40.1, as well as sample 28.2, which is closer to the latter. It should be emphasized that the microstructural parameters obtained from X-ray diffraction provide information about internal structural features but cannot be directly interpreted in terms of sorption performance or surface area in the absence of complementary measurements (e.g., BET surface area and porosity analysis). Therefore, these results are used in the present work to establish systematic trends across the sample series rather than to infer a specific mechanism.

### 2.6. Sorption Equilibria Studies

**Figure 7:**
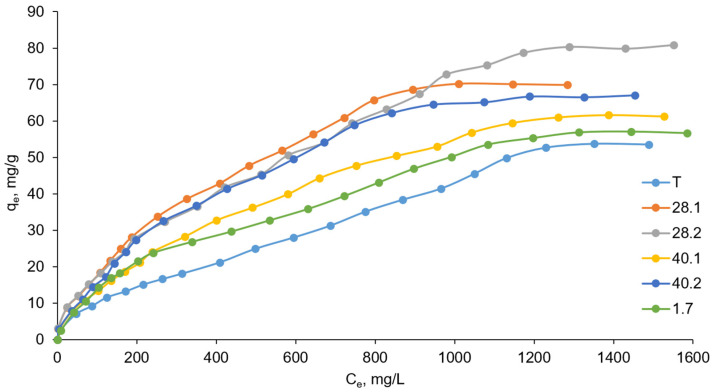
Adsorption isotherms of the prepared LDH (the relative standard deviation for all points was below 5%).

Thus, we prepared LDH using various ultrasonic synthesis modes. To assess the effect of ultrasonic synthesis mode on the adsorption capacity of each prepared LDH, we studied sorption equilibria under isothermal conditions. We used potassium chromate as the adsorbent (hexavalent chromium, an environmentally hazardous pollutant). The resulting adsorption isotherms are shown in Figure 7.

**Figure 8:**
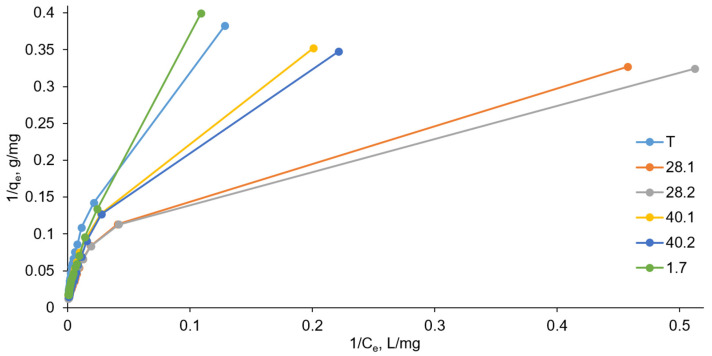
Langmuir models (the relative standard deviation for all points was below 5%).

The obtained results of adsorption experiments were also analyzed using the three most classical models: Langmuir, Freundlich, and Dubinin–Radushkevich. The Langmuir model is the simplest model of so-called monomolecular sorption, which is described by the mathematical equation:

$$q_{e}=\frac{Q_{max}K_{L}C_{e}}{1+K_{L}C_{e}},$$

where C_e_ is the equilibrium concentration of the sorbate, mg/L; q_e_ is the equilibrium amount of sorbate adsorbed per unit weight of the adsorbent, mg/g; K_L_ is the Langmuir constant, L/mg; and Q_max_ is the maximum adsorption capacity, mg/g. The adsorption isotherms in Langmuir coordinates are shown in Figure 8, while isotherm fitting parameters are summarized in Table 8.

The K_L_ value in the Langmuir model characterizes the interaction energy of the sorbate with the sorbent (higher K_L_ values suggest stronger interaction). The K_L_ values were calculated using the equations of the approximating lines as 1/C_e_, at which the adsorbate molecules occupy half of the sorbent’s active adsorption sites (1/C_e1/2_); that is, when q_e_ = Q_max(exper.)_/2. The values of the Langmuir constant K_L_ (Table 8) increase in the LDH series 1.7-T-40.1 and 40.2-28.1-28.2. Thus, the highest interaction between the sorbate and the sorbent according to the Langmuir model is observed in the case of using LDH 28.2. Indeed, the experimental maximum adsorption capacity Q_max(exper.)_ was identified for LDH 28.2. However, the minimum value of Q_max(exper.)_ is characteristic for LDH T, while the Langmuir model suggests the minimum value of the Langmuir constant for LDH 1.7.

Based on the K_L_ values, the Langmuir model allows us to calculate the dimensionless distribution coefficient R_L_:

$$R_{L}=\frac{1}{\left(1+K_{L}C_{0}\right)},$$

where K_L_ is the Langmuir constant, L/mg, and C_0_ is the highest initial sorbate concentration, mg/L. The R_L_ value is very convenient for quantitatively indicating the so-called favorability or unfavorability of a sorption system. The adsorption system is interpreted as favorable when 0 < R_L_ < 1, unfavorable when R_L_ > 1, irreversible when R_L_ = 0, or linear when R_L_ = 1. The distribution coefficient values calculated using the Langmuir model (Table 8) are in the range of 0.0084–0.0644, which indicates favorable adsorption of chromate anions on the synthesized layered double hydroxides, regardless of the use of ultrasound and its mode.

The Langmuir model allows us to calculate the maximum adsorption capacity Q_max(calc.)_ along the segment *a* on the ordinate axis (1/q_e_), which is cut off during extrapolation of the approximating straight line (Q_max(calc.)_ = 1/*a*). Between the calculated data within the Langmuir model and the experimental data, when comparing the maximum adsorption capacity Q_max(calc.)_ and Q_max(exper.)_, we also observe some contradiction. According to the calculated data, the value of Q_max(calc.)_ increases in the LDH series T-40.1-28.1-28.2-40.2-1.7, while the experimentally determined maximum adsorption capacity Q_max(exper.)_ increases in the series T-1.7-40.1-40.2-28.1-28.2.

An inconsistency between the calculated and experimental data, as well as pronounced deviations from linearity in the coordinates 1/q_e_ − 1/C_e_ in some areas, make the Langmuir model quite suitable for describing the adsorption of chromate on the prepared LDH. Langmuir theory describes monomolecular adsorption on a homogeneous solid surface at energetically equivalent active sites in the absence of interaction between the adsorbed particles. The most likely cause of deviations in experimental data from those calculated using the Langmuir model is the surface inhomogeneity of the resulting LDHs. Furthermore, in many cases, the poor applicability of the Langmuir model may be due to the dependence of the adsorption equilibrium constant on the degree of filling, as well as the association of adsorbed particles on the surface or, conversely, their strong repulsion; for example, due to like charges.

**Figure 9:**
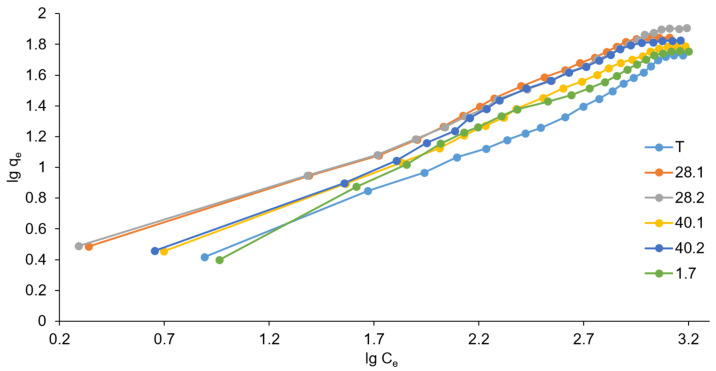
Freundlich models (the relative standard deviation for all points was below 5%).

The data obtained from the study of sorption equilibria were also analyzed within the framework of the Freundlich model (the corresponding isotherms are shown in Figure 9).

The Freundlich model corresponds to the following mathematical equation:

$$lgq_{e}=lgK_{F}+\frac{1}{n}lgC_{e},$$

where q_e_ is the amount of adsorbed sorbate per unit weight of adsorbent, mg/g, K_F_ is a Freundlich constant, mg/g; C_e_ is the equilibrium concentration of the sorbate, mg/L; and 1/n is the heterogeneity index.

The Freundlich model describes the sorption of chromate on the resulting LDHs much better than the Langmuir model, and this is indicated by the regression coefficients R^2^ presented in Table 8. Indeed, the adsorption isotherms in the Freundlich coordinates (Figure 9) have a much more regular linear appearance than in the corresponding Langmuir coordinates (Figure 8).

The Freundlich model assumes that the surface of most adsorbents is heterogeneous, sorbate molecules interact with each other on the surface, and adsorption often does not end with the formation of a monomolecular layer. In this case, the adsorption isotherm equation becomes more complicated. The heterogeneity index 1/n reflects the degree of deviation of the isotherm from a straight line in q_e_ − C_e_ coordinates. Thus, the 1/n index characterizes the heterogeneity of the sorption surface (in the case of a homogeneous surface, 1/n = 1, while with increasing heterogeneity, the 1/n value decreases and approaches 0). According to the data presented in Table 8, the heterogeneity of the LDH surface increases in the series 40.2-1.7-T-40.1-28.1-28.2. Thus, the LDH sample that exhibited the maximum sorption power is characterized by the greatest surface heterogeneity within the framework of the Freundlich model. At the same time, the 1/n parameter represents an empirical descriptor within the Freundlich model and does not provide a direct physical measurement of surface heterogeneity. In the absence of independent characterization methods (e.g., calorimetric measurements or surface charge distribution analysis), this parameter should be interpreted only as a comparative indicator within the studied sample series. Therefore, the observed trend reflects relative differences in adsorption behavior rather than a direct quantification of surface heterogeneity.

The Freundlich constant K_F_ (adsorption capacity) is a quantitative measure of the affinity of the sorbate to the sorbent surface. This parameter reflects the relative sorption capacity of the sorbent (K_F_ corresponds to the q_e_ value at C_e_ = 1). The K_F_ values calculated based on the Freundlich model completely correlate with the experimentally determined values of the maximum sorption capacity Q_max(exper.)_. Both K_F_ and Q_max(exper.)_ increase in the series T-1.7-40.1-40.2-28.1-28.2. Consequently, the Freundlich model is more consistent with experimental data than the Langmuir model.

To describe the adsorption of ions from solutions by porous adsorbents, the Dubinin–Radushkevich model can be effectively used in some cases. The model describes the volumetric filling of the sorbent’s micropores with sorbate. The Dubinin–Radushkevich model corresponds to the following linear equation in coordinates ε^2^ − lnq_e_:

$$lnq_{e}=-K_{D-R}\varepsilon^{2}+ln{Cq}_{s},$$

where q_e_ is the amount of adsorbed sorbate per unit weight of adsorbent; q_s_ is the saturation capacity, mmol/g; K_D − R_ is the Dubinin–Radushkevich constant associated with the free energy of sorption; and ε is the Polyani potential, which is associated with the concentration according to the following equation:

$$\varepsilon =RTln\left(1+\frac{1}{C_{e}}\right),$$

where R is the universal gas constant, 8.314 J/K·mol; T is the absolute temperature, K; and C_e_ is the equilibrium concentration of the sorbate, mg/L.

**Figure 10:**
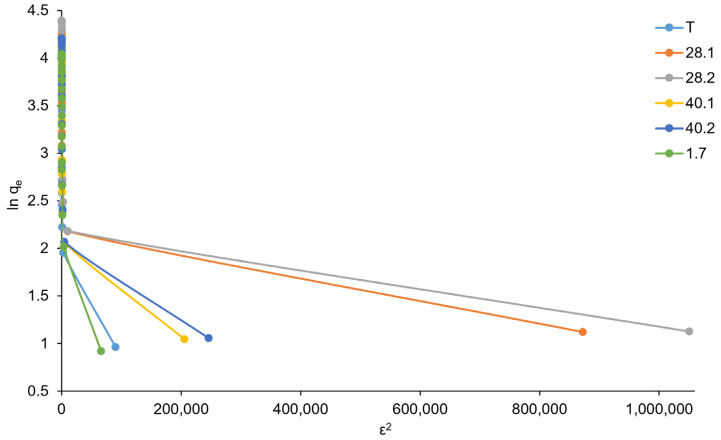
Dubinin–Radushkevich models (the relative standard deviation for all points was below 5%).

However, the isotherm graphs in Dubinin–Radushkevich coordinates (ε^2^ − lnq_e_) shown in Figure 10 demonstrate a pronounced deviation from linearity. Not surprisingly, the low values of the regression coefficients R^2^ (T-0.455, 28.1-0.4521, 28.2-0.4155, 40.1-0.4496, 40.2-0.4443, 1.7-0.5163) indicate the inapplicability of the Dubinin–Radushkevich mathematical model for describing sorption equilibria in the case of chromate anion sorption on the prepared LDHs.

Thus, analysis of the data obtained from the sorption equilibria study revealed that the sorption of chromate anion is satisfactorily described by the Freundlich model. In addition, we can argue that ultrasonic exposure during the preparation of LDH, as well as the synthetic mode of ultrasonic treatment, can influence the sorption properties of synthesized LDHs. The most effective was exposure to ultrasound at a frequency of 28 kHz directly during coprecipitation (sample 28.2), which resulted in an increase in the maximum sorption capacity by 50% in comparison with sample T prepared by the traditional method without any ultrasound action.

The obtained experimental data on the experimental sorption capacity Q_max(exper.)_ correlate well with the obtained characteristics of the microstructure of the studied samples according to the X-ray diffraction data in all three studied models. Sample T, as a rule, has the largest particle sizes and the smallest microdeformations. This combination correlates with its lower sorption performance; however, in the absence of specific surface area (BET) and porosity data, this trend should not be interpreted as direct evidence of a causal relationship. Sample 1.7 is close in its characteristics to sample T and exhibits a similar, but slightly better, sorption capacity (since microdeformations are somewhat larger). This observation is consistent with the general structure–property correlation identified in this study, although the specific contribution of microstrain to sorption remains unresolved. Samples 28.1 and 40.1 are somewhat close to each other. However, 40.1 has slightly larger particles and less microdeformation compared to sample 28.1. These differences are accompanied by moderate variations in sorption capacity, which further support the existence of a correlation between microstructural parameters and sorption behavior. Sample 28.2 turned out to be the leader. It has the smallest particle size and the highest microstrain among the studied samples, and it also exhibits the highest sorption capacity. Importantly, we do not attribute this behavior to a specific mechanism. In particular, although smaller crystallite size is often associated with increased surface area, such a relationship cannot be confirmed in the absence of BET measurements, especially for LDH systems known to form aggregated “sand rose”-like morphologies. Therefore, the observed enhancement in sorption performance may arise from a combination of factors, including possible changes in accessible surface area, increased defect density (microstrain), and/or improved accessibility of interlayer anion exchange sites. The present study does not distinguish between these contributions. As for sample 40.2, despite characteristics similar to sample 28.2, poor crystallization (low crystallinity) of this sample led to average sorption capacity values. This suggests that, in addition to crystallite size and microstrain, the degree of crystallinity also plays an important role in determining sorption performance. This is apparently related to the low heterogeneity of the LDH surface within the Freundlich model (Table 8) for sample 40.2. Sample 28.2, which demonstrated the maximum sorption capacity, is characterized by the greatest surface heterogeneity (Table 8). Thus, the results presented here establish a consistent quantitative correlation between synthesis conditions, microstructural parameters, and sorption properties. However, this correlation should be interpreted as empirical rather than mechanistic and may serve as a basis for future studies aimed at elucidating the underlying physicochemical mechanisms.

### 2.7. Sorption Kinetics Studies

**Figure 11:**
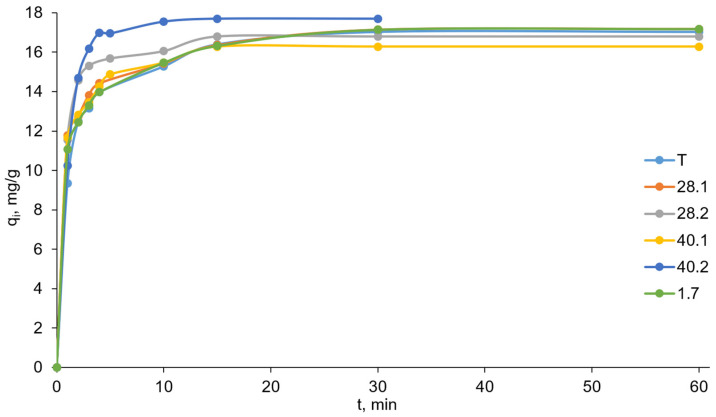
Kinetic curves of chromate anion sorption on synthesized LDHs (the relative standard deviation for all points was below 5%).

For adsorbents (especially in environmental applications), not only is the sorption capacity important but also the sorption process rate. Thus, the next stage of the current study was focused on assessing the kinetics of chromate anion sorption on the prepared LDHs. We sought to understand the effect of ultrasonic treatment during LDH synthesis on the rate of chromate sorption by the synthesized sorbents. To this end, reaction mixtures containing potassium chromate solution and the test sorbent were vigorously stirred at room temperature. At specified time intervals, we collected samples of the reaction mixture and analyzed the loss of chromate. This allowed us to obtain rough data on the dependence of the amount of chromate sorbed on time (Figure 11).

**Figure 12:**
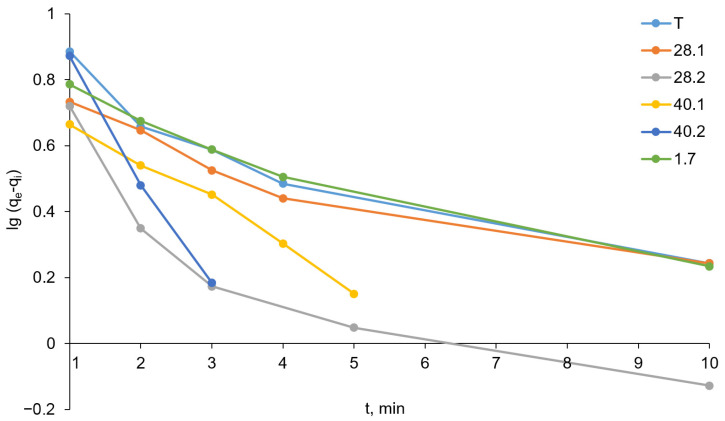
Pseudo-first-order kinetics plots (the relative standard deviation for all points was below 5%).

We analyzed the obtained data using pseudo-first- and pseudo-second-order kinetic models. The linear form of the pseudo-first-order model is described by the following mathematical equation:

$$\mathrm{lg}\left(q_{e}-q_{t}\right)=lgq_{e}-\left(\frac{K_{1}}{2.303}\right)t,$$

where q_t_ and q_e_ are the quantity of sorbate adsorbed on the adsorbent, respectively, at equilibrium and at various times t, mg/g; t is the contact time, min; and K_1_ is the equilibrium rate constant of the pseudo-first-order adsorption process, min^−1^. The pseudo-first-order kinetics plots as ln(q_e_ − q_t_) versus t are shown in Figure 12, and the calculated values of K_1_, q_e_, and the regression coefficient R^2^ are summarized in Table 9.

**Figure 13:**
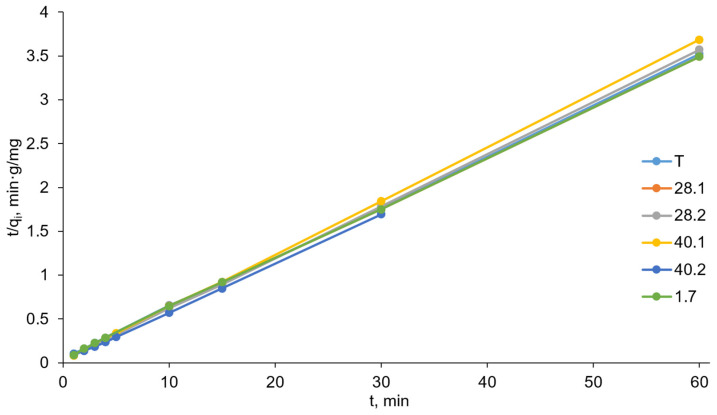
Pseudo-second-order kinetics plots (the relative standard deviation for all points was below 5%).

Figure 13 demonstrates significant deviations from linearity of the pseudo-first-order kinetics plots. The q_e(calc.)_ values calculated from the pseudo-first-order kinetics plots (Table 9) are in almost all cases more than two times smaller than the experimentally determined q_e(exper.)_ values. Only in the case of LDH 40.2 is the q_e(calc.)_ value (15.86 mg/g) sufficiently close to the q_e(exper.)_ value (17.70 mg/g). However, this is not surprising, since only in the case of LDH 40.2 does the pseudo-first-order kinetics plot demonstrate the minimal deviation from linearity and the highest regression coefficient R^2^ = 0.9934 in this series. In general, the low values of the regression coefficients, deviations of the graphs from linearity, and poor agreement between q_e(calc.)_ and q_e(exper.)_ make the pseudo-first-order model poorly suited for describing the kinetics of chromate anion sorption on the prepared LDHs.

The linear form of the pseudo-second-order kinetics model is presented by the mathematical equation:

$$\frac{t}{q_{t}}=\frac{1}{K_{2}q_{e}^{2}}+\left(\frac{1}{q_{e}}\right)\times t,$$

where K_2_ is the equilibrium rate constant of the pseudo-second-order kinetics, g/mg·min; q_t_ and q_e_ are the quantity of sorbate adsorbed on the adsorbent, respectively, at equilibrium and at various times t, mg/g; and t is the contact time, min.

The pseudo-second-order model provides a much better description of the sorption process kinetics. Firstly, the values of regression coefficients R^2^ are very close to 1. Secondly, the q_e(exper.)_ values are in very good agreement with the values calculated from the pseudo-first-order kinetics graphs. For example, for sorbent 28.2, q_e(exper.)_ = 16.80, while q_e(calc.)_ = 16.92. The pseudo-second-order kinetics model allowed us to calculate the rate constants K_2_. The obtained sorbents form the following series in ascending order of K_2_: T-1.7-28.1-40.1-40.2-28.2. Thus, the leading sorbent in terms of sorption rate (as well as sorption capacity) is LDH 28.2.

### 2.8. Characterization of Chromate-Loaded LDH

**Figure 14:**
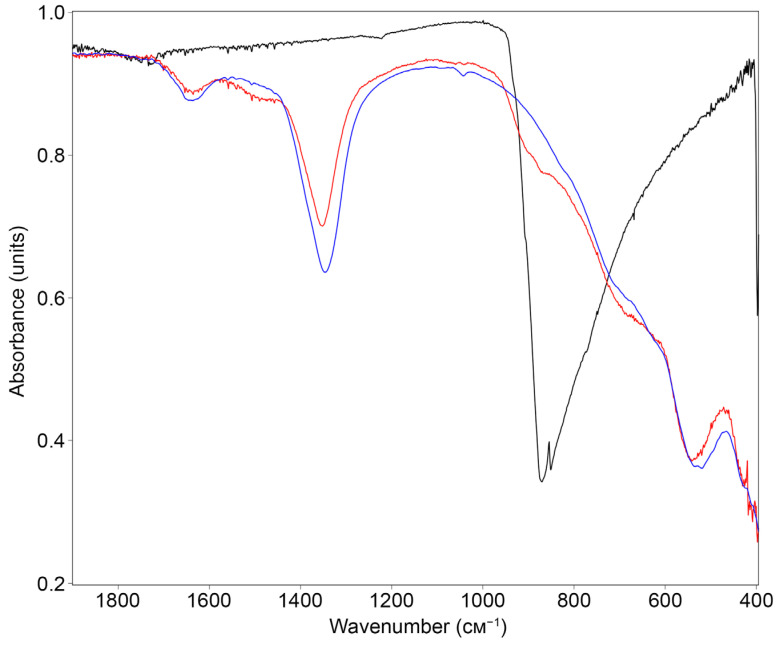
FTIR spectrum of chromate-loaded 28.2 (red), starting 28.2 (blue), and potassium chromate (black).

After chromate sorption, the samples loaded with CrO_4_^2–^ anions were characterized using IR spectroscopy and X-ray diffraction analysis. The IR spectra confirmed the presence of chromate ions in the samples (Figure 14), which appear as a shoulder at approximately 850 cm^−1^ and correspond to the chromate anion vibration band.

**Figure 15:**
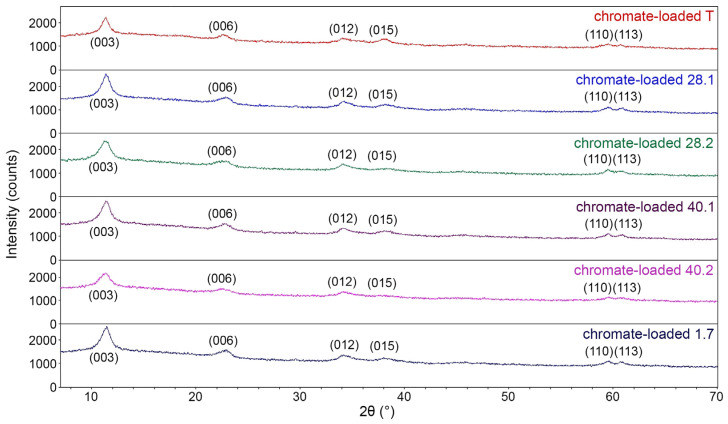
Diffraction patterns of the chromate-loaded LDH.

Figure 15 exhibits the complete diffraction patterns of the synthesized LDH samples after sorption of chromate anions, i.e., in chromate-loaded LDHs. All diffraction patterns are typical for starting unloaded LDH (Figure 5). The diffraction patterns of chromate-loaded LDHs display a slightly reduced noise level and an increased peak intensity-to-noise ratio (compared to similar starting unloaded LDHs). Chromate-loaded LDH 40.2 is again the least crystallized of the other samples. The interlayer distance d_003_ in all LDHs was also 0.77–0.78 nm, although we observed a slight decrease of ~0.001–0.002 nm in most samples. The most pronounced decrease in the interlayer distance (by 0.01 nm) was characteristic of chromate-loaded 28.2. The crystal lattice parameters for chromate-loaded LDHs were a = 3.097–3.102 Å, c = 23.5–23.6 Å, and V = 195–196 Å^3^.

Table 10 shows the crystallite sizes and microstrains in the chromate-loaded LDHs. We obtained these values from X-ray diffraction data using the Scherrer equation and the Stokes and Wilson formula considering the instrumental function, calculated in three approximations: using the full width at half maximum of the peak (FWHM) − FW(S) = β = FWHM with powers N = 1 and N = 2 and the integral broadening (breadth) − FW(S) = β = breadth of single peaks D_003_ and D_006_, as well as their arithmetic mean values D_m_ and regression mean values D_w_ and microstrain ε_w_.

The calculated values of crystallite sizes D and microdeformation ε, although they differ slightly in different models, correlate well. The chromate-loaded LDHs, in comparison with the unloaded species, demonstrate larger particle (crystallite) sizes and smaller microdeformation values (Table 5, Table 6 and Table 10).

X-ray diffraction analysis also shows that after chromate adsorption, the interlayer spacing of LDH remained at 0.77–0.78 nm. Furthermore, loading LDH with chromate anions did not significantly change the unit cell parameters (a and c). Due to the obviously larger size of chromate ions compared to nitrate ions, the interlayer spacing of LDH could have increased after chromate intercalation because of a shift in the (00L) reflections. However, this did not occur. Moreover, the diffraction patterns improved, i.e., the background decreased, the peak-to-background intensity ratio improved, the FWHM and breadth values decreased, and the interlayer values and unit cell parameters decreased slightly after chromate adsorption. Probably, chromate ion adsorption improved and further stabilized the crystal lattice by changing the bond types compared to the more mobile nitrate ions. However, it is also possible that the improved diffraction pattern is simply due to the washing away of amorphous surface phases or better packing of the dried powder after the sorption experiment.

### 2.9. Sorption from a Multicomponent Model Solution

The sorption of chromate ions in multicomponent aqueous systems is typically lower than that observed in deionized water (Table 11). To assess the influence of common inorganic background anions, we performed experiments using a model solution containing chromate (10 mg/L), sulfate (50 mg/L), bicarbonate (100 mg/L), and chloride (100 mg/L). This composition was selected to represent a simplified electrolyte system incorporating major inorganic anions commonly encountered in groundwater environments, rather than a full reproduction of natural groundwater chemistry.

Sorption experiments were conducted by varying the LDH concentration from 0.2 to 1.0 g/L. For comparison, identical experiments were performed in deionized water containing only chromate (10 mg/L), without additional background anions.

The results (Table 11) demonstrate a consistent increase in sorption efficacy with increasing LDH concentration in both systems. As expected, higher removal efficiencies were observed in deionized water, where no competing anions are present. Even at the lowest sorbent concentration (0.2 g/L), chromate removal exceeded 50% for all samples. At higher LDH concentrations (1.0 g/L), complete chromate removal was achieved in deionized water for all materials.

In contrast, the presence of competing anions in the model solution significantly reduced sorption efficiency. At an LDH concentration of 0.2 g/L, the removal efficiency in the multicomponent system was only 27–38% of that observed in deionized water. This behavior is consistent with the known non-selective anion exchange properties of LDHs and the competitive adsorption effects reported in the literature. In particular, sulfate ions, present at a fivefold excess relative to chromate, are known to exhibit higher affinity for LDH interlayers, thereby suppressing chromate uptake [34].

Despite this competitive environment, clear differences between samples remain observable. In particular, sample 28.2 consistently demonstrates the highest sorption efficacy across all tested concentrations. For example, at an LDH concentration of 1.0 g/L, sample 28.2 achieves approximately 68% chromate removal, which is more than twice that of sample T under the same conditions. This trend is consistent with the differences in sorption capacity (Q_max_) discussed above.

Importantly, these results should be interpreted as reflecting sorption behavior in a simplified multicomponent system containing major inorganic anions. Natural groundwater systems may include additional components, such as dissolved organic matter, divalent cations, and trace metals, which could further influence sorption performance. Therefore, the present data provide a controlled basis for comparing material performance under competitive conditions but do not directly represent behavior in real groundwater systems.

Overall, the results indicate that ultrasonic treatment of the synthesis system leads to LDHs with improved resistance to competitive adsorption effects, as evidenced by the superior performance of sample 28.2 in the presence of excess background anions.

### 2.10. Recyclability Studies

**Figure 16:**
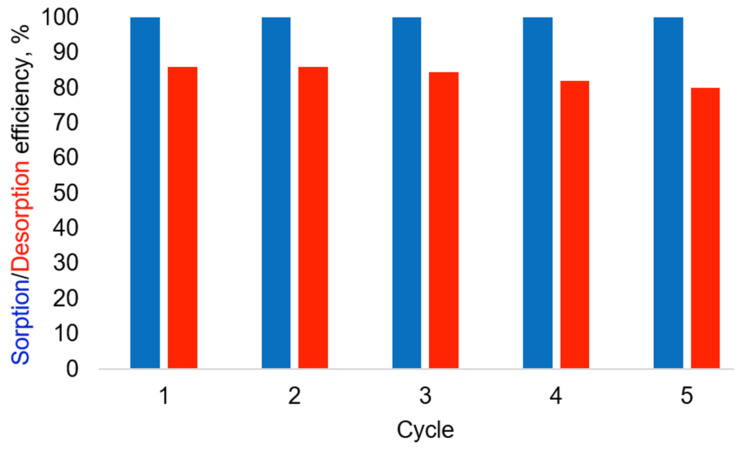
Results of recyclability test (sorption efficacy—blue, desorption efficacy—red). The relative standard deviation for all points was below 5%.

In any case, reusable sorbents always have advantages over disposable ones. Furthermore, reusability is consistent with environmentally friendly principles of green chemistry. Therefore, in this study, we assessed the reusability of the leading LDH (28.2) using a classical adsorption–desorption test. In our study, each test cycle involved immersing the test sorbent (LDH 28.2, 1 g/L concentration) in a chromate solution (10 mg/L). The reaction mixture was maintained at room temperature for 2 h, after which we detected the adsorption efficiency by measuring the loss of chromate from the reaction mixture. A desorption experiment was then conducted, which consisted of treating the chromate-loaded sorbent with a sodium hydroxide solution (1 mol/L) for 2 h. This allowed us to detect the amount of chromate released into the sodium hydroxide solution and evaluate the desorption efficiency. We conducted five such cycles. The experimental results are shown in Figure 16.

Desorption efficiency in the series of sequential cycles 1–5 decreased slightly and reproducibly, from 86% in the first cycle to 80% in the fifth cycle. The relative standard deviation for all points was below 5%; however, no separate statistical significance test was performed for differences between individual cycles. For example, desorption efficiency in the first cycle is 86%, while by the fifth cycle, we observed a decrease to 80%. Despite a slight decrease in desorption efficiency (approximately 6%), adsorption efficiency in all five cycles remains close to quantitative removal under the applied conditions, which indicates that the system operates far below the maximum sorption capacity of the material. It should be noted that the initial chromate concentration (10 mg/L) is significantly lower than the maximum sorption capacity (Qmax) of the LDH, as estimated from the isotherm data. Therefore, even partial retention of chromate after desorption does not lead to a noticeable decrease in adsorption efficiency under these experimental conditions, since the number of available sorption sites remains sufficient.

This is consistent with the conditions used in the sorption isotherm experiments, where the system approached saturation at significantly higher chromate-to-sorbent ratios (e.g., 25 mg of chromate per 0.15 g of LDH in 12 mL H_2_O), whereas in the present recyclability experiments, a much lower loading is used (10 mg/L chromate and 1.00 g LDH in 1000 mL H_2_O). Thus, the recyclability tests are carried out under conditions far from saturation.

It is also worth noting that LDH 28.2 is virtually resistant to metal leaching under the influence of the sodium hydroxide solution (1 mol/L) used for desorption. We monitored the concentration of Mg^2+^ and Al^3+^ cations during recyclability tests using inductively coupled plasma mass spectrometry and confirmed that the total leaching of metal ions by the fifth cycle is no more than 3% relative to the initial LDH. At the same time, it should be emphasized that the present recyclability test was carried out under conditions far from sorbent saturation and, therefore, does not allow direct evaluation of capacity loss at higher loadings. All these observations encouraged us to consider LDH 28.2 as an effective and reusable sorbent under the applied laboratory conditions, while further studies at higher loadings are required for a complete assessment of long-term performance.

### 2.11. Sorption of Cr(VI) from Soil

Soil experiments were conducted using a controlled laboratory model system to evaluate the ability of LDH materials to reduce the water-soluble fraction of hexavalent chromium under comparable conditions. It should be noted that the applied contamination protocol (spiking with chromate solution followed by 48 h equilibration) represents a simplified approach and does not reproduce the complexity of aged, field-contaminated soils, where chromium may be associated with mineral phases (e.g., iron oxides) or organic matter.

In the preliminary stage, the optimal eluent for leaching hexavalent chromium from contaminated soil was determined. Soil samples (15 g) were spiked with potassium chromate solution and equilibrated for 48 h in closed vessels to prevent drying. The contaminated soil samples were then washed with different eluents: (i) deionized water acidified with sulfuric acid to pH 4.5, (ii) an acetate–ammonia buffer solution at pH 4.5, and (iii) a nitric acid solution (1 mol/L). The highest chromium recovery in the eluate was observed when using deionized water acidified with sulfuric acid to pH 4.5, and this eluent was, therefore, selected for subsequent experiments.

In the next step, soil samples were contaminated with varying amounts of chromate (3.30–91.65 mg Cr(VI), corresponding to 0.75–21 mL of a 0.25 N potassium chromate solution diluted to 25 mL). Parallel experiments were performed with the addition of LDH (sample 28.2, 3 wt.% relative to soil), introduced as a suspension in 5 mL of deionized water. Control samples were prepared in the same way without LDH.

After equilibration, all samples were subjected to extraction using the selected eluent (aqueous H_2_SO_4_, pH 4.5). The extraction procedure consisted of three successive cycles with fresh portions of eluent. The combined eluates were concentrated by evaporation and analyzed for chromium content. The experimental data are presented in Table 12.

The results indicate that, in the absence of LDH, soil retains a significant portion of added chromium, with only a fraction present in the water-soluble form. For example, at the highest contamination level (91.65 mg Cr(VI)), only 14.31 mg was detected in the eluate, corresponding to approximately 16% of the total chromium content. This fraction represents the mobile and potentially bioavailable form of chromium.

The addition of LDH (sample 28.2) resulted in a substantial decrease in the chromium content in the eluate. Under identical contamination conditions (91.65 mg Cr(VI)), only 0.26 mg of chromium was detected after extraction, indicating effective immobilization of chromate in the soil matrix under the applied conditions.

**Figure 17:**
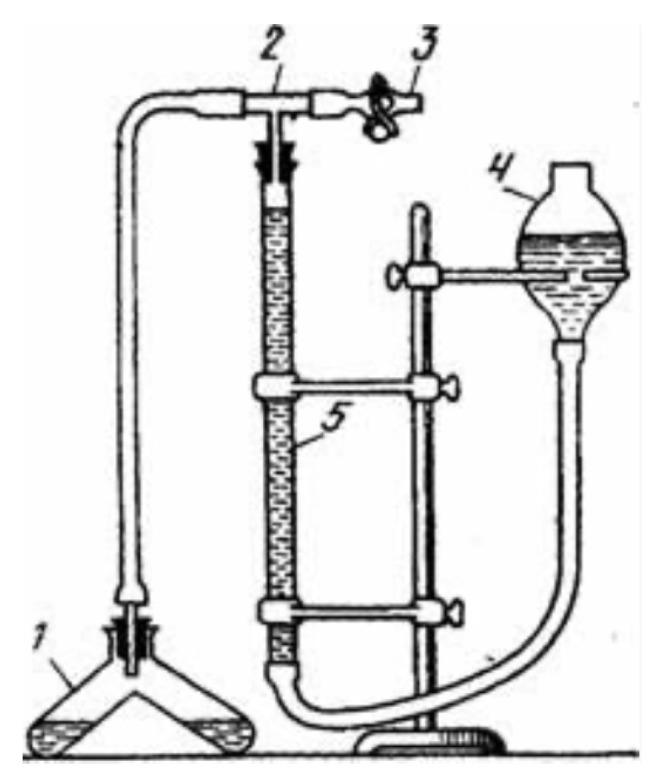
Gas-volumetric determination of catalase activity of soils (1—reservoir for soil and hydrogen peroxide solution, 2—glass tube, 3—valve, 4—reservoir with water, 5—graduated burette for cm^3^ O^2^).

The biological impact of chromate was assessed using soil catalase activity as a general indicator of oxidative stress. Catalase catalyzes the decomposition of hydrogen peroxide, and its activity can be quantified by measuring the volume of oxygen released per unit time and soil mass. Catalase activity was determined using a gas-volumetric method based on oxygen evolution (Figure 17), in which 5.00 g of soil was reacted with 10.00 mL of a 3% hydrogen peroxide solution, and the released oxygen volume was measured after 5 min. The method is consistent with previously reported catalase-related assays based on oxygen evolution and gasometric measurements [35,36].

It is well established that soil catalase activity depends on soil composition and environmental factors and represents a non-specific indicator of biological activity rather than a selective measure of chromium toxicity [37]. Therefore, in the present study, catalase activity was used as a comparative proxy under identical experimental conditions.

In uncontaminated soil, catalase activity was approximately 0.7 cm^3^ O_2_ g^−1^ min^−1^. In soil samples contaminated with chromate and not treated with LDH, catalase activity was strongly suppressed and was nearly completely inhibited at moderate and high contamination levels. In contrast, the addition of LDH significantly mitigated this effect, and catalase activity was largely preserved even at high chromium loadings (with only a moderate decrease at the highest contamination level).

These results indicate that, under the applied laboratory conditions, LDH addition reduces the water-soluble fraction of chromate and mitigates its inhibitory effect on soil enzymatic activity. However, it should be noted that LDH materials are basic and may alter the pH of the soil system, which can also influence chromium mobility and enzyme activity. Therefore, the observed effects may arise from a combination of sorption and pH-related factors, and their individual contributions were not decoupled in the present study. Thus, these findings should be interpreted within the limitations of the simplified experimental system, and further studies are required to assess behavior in more complex and realistic soil environments.

## 3. Materials and Methods

### 3.1. Materials

Magnesium(II) nitrate hexahydrate, iron(III) nitrate nonahydrate, sodium hydroxide, sodium nitrate, potassium chromate, 1,5-diphenylcarbazide, sulfuric acid, catalase, and hydrogen peroxide were purchased from Sigma-Aldrich (St. Louis, MO, USA). Acetate–ammonia buffer solution and nitric acid were obtained from Lenreactiv (Saint Petersburg, Russia). Other chemicals, solvents, and materials were obtained from commercial sources and used without any additional purification.

### 3.2. Synthesis of LDH

Sodium hydroxide (27 g) was dissolved in 300 mL of deionized water (solution A). Magnesium(II) nitrate hexahydrate (26 g) and iron(III) nitrate nonahydrate (20 g) were dissolved in 200 mL of deionized water (solution B). Solution A was added dropwise to solution B for 15 min under stirring, and the reaction was kept for 24 h at 70 °C (sample T). Solution A was dropwise added to solution B during 15 min under stirring, and then the reaction was treated by ultrasound (28, 40 kHz, or 1.7 MHz) for 15 min (samples 28.1, 40.1, and 1.7). Solution A was dropwise added to solution B for 15 min under stirring and simultaneous ultrasonication (28 or 40 kHz) for 15 min (samples 28.2 and 40.2). The pH was maintained at 13 during the synthesis. The reaction mixtures were centrifuged at 8000 rpm. The brown precipitate was washed with deionized water until the pH reached a neutral value and the qualitative reaction for nitrate ion disappeared. The obtained purified LDHs were dried at 70 °C to constant weight. The samples were analyzed for magnesium and iron content using inductively coupled plasma mass spectrometry.

The ultrasonic device is a Digital Pro^®^ (Shenzhen Guan Yijia Technology Co., Ltd., Shenzhen, China) ultrasonic bath, with a nominal power of 150 W. The volume of the treated medium (water) was 2000 mL, the position of the reaction vessel in the ultrasonic bath is centered and fixed, and the exposure time is 15 min.

*Limitations.* In this study, ultrasonic treatment was performed using an ultrasonic bath, which, unlike ultrasonic horn systems, does not allow direct measurement of the end-face oscillation amplitude, gain, or local specific power within the volume of the treated medium. Energy is transferred through the liquid within the bath, creating a complex, non-uniform cavitation field. However, the conditions and results of ultrasonic treatment were reproducible in all experiments, allowing for their accurate comparison within this study. The ultrasonic power applied to the liquid was estimated calorimetrically using the equation:

P = m c ΔT/Δt,

where m is the mass of the liquid (kg), c is the specific heat capacity (J/kg K), and Δt/ΔT is the average heating rate (K/s). The resulting value was approximately 63.6 W, corresponding to a specific power of approximately 0.032 W cm^−3^. However, it should be noted that the obtained value includes heat loss and is averaged over the entire volume of the medium (water).

### 3.3. Characterization

Thermogravimetric analysis was carried out on a TA Instruments (New Castle, DE, USA) TGA Q500 device, under a nitrogen atmosphere, using a heating rate of 5 °C per minute within a temperature spectrum of 30 °C to 850 °C.

Fourier-transform infrared spectra (FTIR) were recorded in an Infraspec (Saint Petersburg, Russia) FSM 2202 infrared Fourier spectrometer with attenuated total-reflectance mode in the range of 500–4000 cm^−1^. A total of 8 scans were performed with a resolution of 4 cm^−1^.

X-ray diffraction analysis of the prepared LDH was carried out on a Tongda (Dandong Tongda Science & Technology Co., Ltd., Dandong, China) TD-3700 diffractometer (40 kV and 30 mA, CuKα radiation, Ni filter, a high-speed one-dimensional array detector) at room temperature and a θ/θ scan in the range from 7° to 70° 2θ. X-ray diffraction peak profiles were approximated using the pseudo-Voigt function. The profile characteristics were refined: peak position, its intensity, the full width at half maximum of the peak, breadth, etc. The refinement quality was controlled using statistical criteria. To determine the physical broadening function (considering instrumental broadening), polycrystalline Si powder annealed at 300 °C for 24 h was used as a standard.

The microstructure study (SEM) was carried out using a Tescan (Tescan Group, Brno, Czech) Amber GMH scanning microscope (Kurnakov Institute of General and Inorganic Chemistry) at 1.00 kV, an SE2 detector, a working distance of 5.00 mm, and a pressure of 1.2 × 10^−6^ mbar. For SEM analysis, a small amount of dry LDH powder was placed onto conductive carbon adhesive tape mounted on an aluminum SEM stub. Excess powder was removed by a gentle gas flow. No additional chemical treatment or dispersion procedure was applied. SEM imaging was performed at a low accelerating voltage to minimize charging effects.

The magnesium to iron mass ratio was analyzed by inductively coupled plasma mass spectrometry on a NexION 300D instrument (PerkinElmer Inc., Shelton, CT, USA). The mass ratio of nitrate ions in relation to the found mass ratio of magnesium and iron was determined by reverse dichromatometric titration as described elsewhere [24]. Briefly, an excess of a standard iron(II) solution was added to a solution containing nitrate ions, prepared by dissolving a suspension of LDH in concentrated sulfuric acid. After completion of the reaction, the remaining iron was titrated with a standard sodium dichromate solution.

### 3.4. Sorption Experiments

For sorption equilibria study, 0.60 mL 0.0125 N, or 1.00, 1.50, 2.00, 2.50, 3.00, 3.50, 4.00, 5.00, 6.00, 7.00, 8.00, 9.00, 10.00, and 11.00 mL 0.0250 N, or 6.00, 6.50, 7.00, 7.50, and 8.00 mL 0.0500 N solution of potassium chromate was mixed with deionized water to a general volume of 12.00 mL in a reaction vial. Each vial was charged with 0.150 g of tested sorbent, sealed, and shaken at 20 °C for 24 h. The reaction mixtures were centrifuged for 3 min at 8000 rpm, and the supernatants were analyzed for chromate content.

For the kinetics study, a reaction flask containing 150.00 mL of aqueous solution containing 3.00 mg of potassium chromate was charged with a tested sorbent (0.150 g), sealed, and shaken at 25 °C. We collected samples (5 mL) with a syringe filter at 1, 2, 3, 4, 10, 15, 30, and 60 min after the start of the experiment and analyzed them for chromate content.

### 3.5. Experiments with the Multicomponent Model Solution

The qualitative and quantitative composition of the principal ions of natural groundwater from the Ulanovichi district of Vitebsk, Republic of Belarus, was used as the basis for the multicomponent model solution. To prepare the solution, we dissolved calcium chloride dihydrate (207 mg), magnesium sulfate heptahydrate (128 mg), and sodium bicarbonate (141 mg) per 1 L of deionized water. Both the multicomponent model solution and deionized water were contaminated with chromate, which was dissolved at a concentration of 16 mg per 1 L. A total of 0.2, 0.5, or 1.0 g of LDH 28.2 sorbent was added to 1.00 L of the chromate-contaminated multicomponent model solution or deionized water, and the mixture was gently stirred at 30 rpm at room temperature for 2 h. The chromate content in the water was then estimated.

### 3.6. Recyclability Assessment

A solution with a chromate anion concentration of 10 mg/L was obtained by dissolving potassium chromate in deionized water at a ratio of 16 mg potassium chromate to 1.00 L of water. A total of 0.500 g LDH 28.2 was suspended in the resulting chromate solution (500 mL) and stirred at room temperature for 2 h, after which the concentration of chromate anions in the solution (adsorption efficiency) was estimated. Then, LDH loaded with chromate anions was centrifuged. After centrifugation, the supernatant was collected in a separate vessel, and LDH was suspended in 500 mL of sodium hydroxide solution (1 mol/L) and stirred for 2 h at room temperature. The mixture was centrifuged, the supernatant was collected, and the concentration of chromate anions (desorption efficiency) was estimated. Five sorption–desorption cycles were carried out in this manner. At the end of the experiments, all supernatants were pooled, evaporated, and analyzed for magnesium and iron content using inductively coupled plasma mass spectrometry.

### 3.7. Soil Experiments

A suspension of 0.3 g LDH in 5 mL water was added to 15 g of air-dried soil and mixed thoroughly. Control soil samples were similarly supplemented with 5 mL of water without any LDH. A total of 25 mL of a solution obtained by bringing 0.75, 1.5, 3, 6, 12, 15.75, and 21 mL of a 0.25N potassium chromate solution to 25 mL with water was added to the prepared soil samples. The samples were kept at room temperature for 48 h and then sealed to prevent drying out. Then, 200 mL of eluent (deionized water acidified with sulfuric acid to pH 4.5) was added to the samples, and the resulting mixture was shaken at room temperature for 2 h. The extraction cycle was repeated three times with the same new portions of eluent. The eluates were combined, evaporated, and analyzed for chromium content.

For the catalase activity study, the apparatus shown in Figure 17 was used.

Reservoir 4 was filled with water, bringing the water level through a system of communicating vessels to the “0” mark on graduated burette 5. Valve 3 was set to the “closed” position. One compartment of reservoir 1 was filled with the test soil sample (5.00 g), and the other compartment was charged with a 3% aqueous hydrogen peroxide solution (10.00 mL). Reservoir 1 was connected to glass tube 2 and rotated to completely transfer the hydrogen peroxide solution into the compartment containing the soil sample. At this point, the stopwatch was started, and after 5 min, the volume of oxygen released was recorded in graduated burette 5. The experiments were performed three times for each soil sample.

### 3.8. Statistics

Experimental data were processed using descriptive statistics. Reproducibility was evaluated using the relative standard deviation (RSD), which was below 5% for the experimental points.

## 4. Conclusions

The results of this work can be considered from the following few perspectives.

First, we prepared and fully characterized Mg/Fe LDHs using different ultrasonic synthesis modes, i.e., 28 and 40 kHz (samples 28.1 and 40.1) or 1.7 MHz (sample 1.7) after coprecipitation and 28 or 40 kHz (samples 28.2 or 40.2) during coprecipitation. The ultrasonic treatment time was 15 min, while the conventional synthesis (sample T) required 24 h of thermal treatment. Thus, the ultrasonic approach allows for a substantial reduction in synthesis time under the conditions used in this work.

Second, X-ray diffraction studies showed that the applied synthetic ultrasound modes are accompanied by systematic differences in the microstructural parameters of the prepared LDHs, including crystallite size and microstrain. The largest crystallite sizes and the smallest microstrains are characteristic of the samples prepared by conventional synthesis (T) or obtained by ultrasound after coprecipitation (40.1 and 1.7). Sample 28.1 occupies an intermediate position, whereas ultrasonic treatment during coprecipitation (28.2 and 40.2; sample 40.2 being poorly crystallized) leads to smaller coherent scattering domains and higher microstrain. These XRD-derived parameters describe coherent scattering domains and lattice distortions and should not be directly interpreted as accessible surface area or porosity in the absence of BET data. Therefore, the observed microstructural trends are treated here as empirical structure-related characteristics rather than as proof of a specific mechanism.

Third, all synthesized LDHs sorbed chromate anions, and the sorption kinetics were better described by the pseudo-second-order model. The obtained sorbents form the following series in ascending order of K_2_: T-1.7-28.1-40.1-40.2-28.2. Thus, LDH 28.2 is the leading sample in terms of sorption rate under the applied conditions. The Freundlich model provides a better description of the sorption equilibria than the Langmuir and Dubinin–Radushkevich models. Both the Freundlich constant KF and the experimentally determined Q_max_ increase in the series T-1.7-28.1-40.1-40.2-28.2. These trends correlate with the XRD-derived microstructural parameters; however, this correlation should be interpreted as empirical rather than mechanistic. The present study does not distinguish between possible contributions from accessible surface area, defect density, interlayer accessibility, or surface charge effects.

Fourth, chromate adsorption did not significantly alter the unit-cell parameters or the LDH interlayer spacing. After chromate adsorption, the diffraction patterns showed reduced background, improved peak-to-background ratio, and decreased FWHM and peak breadth values. These changes may indicate partial stabilization or ordering of the LDH structure after chromate uptake; however, alternative explanations such as removal of amorphous surface phases during washing or improved packing of the dried powder after the sorption experiment cannot be excluded.

Fifth, LDH 28.2 demonstrated the best performance in additional application-oriented tests. In a simplified multicomponent model solution containing major inorganic background anions, sample 28.2 showed the highest chromate removal among the tested LDHs, although these experiments should not be considered a direct simulation of real groundwater. Recyclability experiments showed approximately 80% chromate desorption under alkaline conditions and near-quantitative chromate removal over five cycles under conditions far from sorbent saturation. Therefore, these results demonstrate reusability under the applied laboratory conditions but do not directly quantify capacity loss at higher chromate loadings.

Finally, controlled soil experiments showed that LDH 28.2 reduces the water-soluble fraction of Cr(VI) and mitigates the inhibitory effect of chromate on soil catalase activity under the applied laboratory conditions. However, these experiments represent a simplified model system rather than aged, field-contaminated soil. Moreover, catalase activity was used only as a non-specific comparative proxy for soil biological activity, and the possible contribution of LDH-induced pH changes was not decoupled from the sorption/immobilization effect. Overall, the present study demonstrates that ultrasound applied at different stages of Mg/Fe LDH synthesis can systematically affect XRD-derived microstructural parameters and chromate sorption performance, while further mechanistic studies, including surface area, porosity, pH-resolved, and real-matrix experiments, are required to fully elucidate the underlying processes and practical applicability.

## Figures and Tables

**Table 1:** Ultrasound and magnesium–iron LDHs.

Entry	Reference	Ultrasound Application Stage	Area of Application	Frequency/Power
**1**	[21]	after coprecipitation	water remediation (sorption of nitrate, rifampicin, 17-α-methyltestosterone)	NA/NA *
**2**	[22]	before and during coprecipitation	sorption (phosphorus recovery)	NA/NA
**3**	[23]	after coprecipitation	water remediation (chromate sorption)	22 kHz/NA
**4**	[24]	after coprecipitation	water remediation (chromate sorption)	22 kHz/NA
**5**	[15]	during in vivo use	biomedicine	19.876 MHz/NA
**6**	[14]	during in vivo use in sonodynamic therapy	LDH-promoted generation of ROS (•O_2_^−^, ^1^O_2_) under ultrasonic irradiation	30 kHz/NA
**7**	[13]	at the composite preparation stage	tissue regeneration	NA/NA
**8**	[25]	before and during coprecipitation	CO_2_ sorption	NA/NA
**9**	[26]	during use in catalytic reaction	1,3-dipolar cycloaddition	37 kHz/320 W
**10**	[27]	during coprecipitation	uranium(VI) removal	50–60 kHz/1.0 W/cm^2^
**11**	[28]	during sorption process	perchlorate removal	40 kHz/NA
**12**	[29]	during coprecipitation	Reactive Orange 16 and Crystal Violet dyes sorption	NA/NA
**13**	[30]	before coprecipitation and during composite preparation stage	photocatalytic degradation of chloramphenicol	NA/NA

* NA—no answer.

**Table 2:** Conditions for the synthesis of layered double hydroxides and their code names and Mg/Fe ratio.

LDH, Mg/Fe Ratio	Heating		Ultrasonic Treatment		
	Temperature, °C	Time, min	Order of Application	Frequency, kHz	Time, min
**T, *2.05***	70	1440	–	–	–
**28.1, *1.96***	–	–	after coprecipitation	28	15
**28.2, *2.07***	–	–	during coprecipitation	28	15
**40.1, *1.93***	–	–	after coprecipitation	40	15
**40.2, *2.06***	–	–	during coprecipitation	40	15

**Table 3:** Characteristic groups and corresponding wave numbers in IR spectra of the synthesized LDHs.

LDH	ν(O–H), cm^−1^	δ(H_2_O), cm^−1^	ν(N–O), cm^−1^	ν(M–O), cm^−1^
**T**	3398	1645	1347	521
**28.1**	3400	1643	1347	515
**28.2**	3398	1642	1346	519
**40.1**	3395	1642	1346	518
**40.2**	3399	1647	1343	520
**1.7**	3398	1645	1346	521

**Table 4:** Key parameters of the thermal decomposition stages of the synthesized LDHs.

LDH	The 1st Stage of Thermal Decomposition		The 2nd Stage of Thermal Decomposition		The 3rd Stage of Thermal Decomposition	
	T_1max_, °C	Weight Loss, %	T_2max_, °C	Weight Loss, %	T_3max_, °C	Weight Loss, %
**T**	126	11	336	17	461	5
**28.1**	123	12	341	16	460	4
**28.2**	123	12	339	17	456	5
**40.1**	125	11	336	18	458	5
**40.2**	121	12	342	18	456	5
**1.7**	123	12	341	18	458	6

**Table 5:** Calculated values of crystallite sizes based on the profiles of single reflection peaks (003) and (006), taking into account the instrumental function for T, 28.1, 28.2, 40.1, 40.2, and 1.7.

Crystallite Size	LDH					
	T	28.1	28.2	40.1	40.2	1.7
	**FW(S) = β = FWHM (n = 1)**					
**D_003_, Å**	78	72	63	80	61	76
**D_006_, Å**	61	56	46	55	48	59
**D_m_, Å**	70	64	55	68	55	68
**D_w_, Å**	76	70	61	77	60	75
	**FW(S) = β = FWHM (n = 2)**					
**D_003_, Å**	71	66	58	73	57	70
**D_006_, Å**	57	52	44	55	45	55
**D_m_, Å**	64	59	51	64	51	63
**D_w_, Å**	70	65	57	71	56	69
	**FW(S) = β = Breadth**					
**D_003_, Å**	54	51	45	54	43	54
**D_006_, Å**	44	44	36	43	35	42
**D_m_, Å**	49	48	41	49	39	48
**D_w_, Å**	53	51	44	53	42	53

**Table 6:** Calculated values of microdeformations based on the profiles of single reflection peaks (003) and (006), taking into account the instrumental function for samples T, 28.1, 28.2, 40.1, 40.2, and 1.7.

Microdeformations	LDH					
	T	28.1	28.2	40.1	40.2	1.7
	**FW(S) = β = FWHM (n = 1)**					
**ε_w_, 10^−2^**	4.06	4.46	5.19	4.01	5.28	4.23
	**FW(S) = β = FWHM (n = 2)**					
**ε_w_, 10^−2^**	4.42	4.83	5.57	4.36	5.65	4.61
	**FW(S) = β = Breadth**					
**ε_w_, 10^−2^**	5.84	6.12	7.16	6.07	7.5	5.99

**Table 7:** Values of the microstructure parameters of samples T, 28.1, 28.2, 40.1, and 1.7, calculated by the Williamson–Hall method [33] based on the reflection profiles (003) and (006), taking into account the instrumental function.

Characteristics	LDH				
	T	28.1	28.2	40.1	1.7
	**FW(S) = β = FWHM (n = 1)**				
**D, Å**	106	101	98	105	107
**ε, 10^−2^**	1.281	1.386	2.031	1.372	1.314
	**FW(S) = β = FWHM (n = 2)**				
**D, Å**	95	90	88	93	96
**ε, 10^−2^**	1.316	1.644	2.066	1.421	1.348
	**FW(S) = β = Breadth**				
**D, Å**	69	63	58	65	70
**ε, 10^−2^**	1.428	1.556	1.798	1.516	1.474

**Table 8:** Isotherm fitting parameters of CrO_4_^2–^ adsorption.

LDH	Q_max(calc.)_, mg/g	Langmuir Model				Freundlich Model		
		Q_max(exper.)_, mg/g	K_L_, L/mg	R_L_	R^2^	K_F_, mg/g	1/n	R^2^
**T**	25.126	53.70	0.0144	0.0355	0.9407	0.6812	0.5905	0.9874
**28.1**	30.864	70.23	0.0492	0.0107	0.9167	1.6304	0.5423	0.9895
**28.2**	33.112	80.35	0.0513	0.0084	0.9050	1.6823	0.5331	0.9888
**40.1**	30.488	60.99	0.0199	0.0242	0.9351	1.0062	0.5770	0.9970
**40.2**	33.784	66.71	0.0199	0.0242	0.9314	1.0287	0.6035	0.9923
**1.7**	38.910	56.84	0.0073	0.0644	0.9864	0.7903	0.6024	0.9913

**Table 9:** Kinetic parameters of chromate sorption by the synthesized LDHs.

LDH	q_e(exper.)_, mg/g	Pseudo-First-Order Kinetics			Pseudo-Second-Order Kinetics		
		K_1_, min^−1^	q_e(calc.)_, mg/g	R^2^	K^2^, g/mg·min	q_e(calc.)_, mg/g	R^2^
**T**	17.03	0.1428	6.60	0.8682	0.0630	17.33	0.9999
**28.1**	17.17	0.1177	5.27	0.9071	0.0715	17.42	0.9998
**28.2**	16.80	0.1806	3.65	0.7459	0.1681	16.92	1.0000
**40.1**	16.28	0.2909	6.33	0.9908	0.1197	16.44	0.9999
**40.2**	17.70	0.7924	15.86	0.9934	0.1424	18.02	0.9997
**1.7**	17.17	0.1384	6.15	0.9598	0.0640	17.45	0.9999

**Table 10:** Values of the microstructure parameters: crystallite sizes and microdeformation in chromate-loaded LDH calculated from the profiles of single reflection peaks (003) and (006) considering the instrumental function.

Characteristics	FW(S) = β = FWHM (n = 1)	FW(S) = β = FWHM (n = 2)	FW(S) = β = Breadth
**Crystallite Size**			
**D_003_, Å**	103–69	90–64	70–49
**D_006_, Å**	74–47	66–45	53–37
**D_m_, Å**	89–58	78–55	62–43
**D_w_, Å**	100–66	87–61	68–48
**Microdeformation**			
**ε_w_, 10^−2^**	3.07–4.71	3.51–5.07	4.46–6.45

**Table 11:** The results of simplified electrolyte system experiments (the relative standard deviation for all points was below 5%).

LDH	Water			Simulated Groundwater		
	LDH Concentration, g/L					
	0.2	0.5	1.0	0.2	0.5	1.0
	Sorption Efficacy, %					
**T**	52	78	100	14	21	30
**28.1**	63	84	100	19	26	41
**28.2**	68	89	100	26	39	68
**40.1**	60	82	100	18	28	40
**40.2**	60	80	100	20	28	41
**1.7**	54	81	100	16	20	34

**Table 12:** Effect of LDH on the Cr(VI) content in the eluate and on the catalase activity of the soil (the relative standard deviation for all points was below 5%).

Entry	Soil Contamination with Cr(VI), mg/g	Content of Cr(VI) in Eluate, mg	Catalase Activity, cm^3^/g/min
**1**	3.30	0	0
**2**	6.60	0.70	0
**3**	13.20	1.39	0
**4**	26.40	2.22	0
**5**	52.80	4.53	0.11
**6**	69.30	9.19	0.18
**7**	91.65	14.31	0.26
**8**	0	0	0

## Data Availability

The original contributions presented in this study are included in the article. Further inquiries can be directed to the corresponding author.

## References

[1] Kameliya J., Verma A., Dutta P., Arora C., Vyas S., Varma R.S. (2023). Layered Double Hydroxide Materials: A Review on Their Preparation, Characterization, and Applications. Inorganics.

[2] Mishra G., Dash B., Pandey S. (2018). Layered double hydroxides: A brief review from fundamentals to application as evolving biomaterials. Appl. Clay Sci..

[3] Khan A.I., O’Hare D. (2002). Intercalation chemistry of layered double hydroxides: Recent developments and applications. J. Mater. Chem..

[4] Liu Y., Jiang S., Xu J. (2022). Exploring the intercalation chemistry of layered yttrium hydroxides by ^13^C solid-state NMR spectroscopy. Magn. Reson. Lett..

[5] Altalhi A.A., Mohamed E.A., Negm N.A. (2024). Recent advances in layered double hydroxide (LDH)-based materials: Fabrication, modification strategies, characterization, promising environmental catalytic applications, and prospective aspects. Energy Adv..

[6] Johnston A.L., Lester E., Williams O., Gomes R.L. (2021). Understanding Layered Double Hydroxide properties as sorbent materials for removing organic pollutants from environmental waters. J. Environ. Chem. Eng..

[7] Flores C.V., Obeso J.L., Herrera-Zuñiga L., Peralta R.A., Campero-Domínguez J.I., Morales-Ruiz L., Portillo-Vélez N.S., Valdivia-Corona J.C. (2026). Layered double hydroxides (LDH) materials for effective phosphate adsorption from aqueous solution. RSC Sustain..

[8] Sheikhzade M.H., Moghaddam A., Khonakdar H.A., Safarkhani M., Zare E.N., Huh Y., Maleh H.K. (2026). Toward layered double oxide-based nanomaterials: From synthesis to diverse applications. Coord. Chem. Rev..

[9] Lv T.Y., Cui Z.L., Guo R.T. (2026). Application of layered double hydroxides (LDHs) derived catalysts for selective catalytic reduction of NOx with NH_3_. Fuel.

[10] Astafiev A.A., Shakhov A.M., Kritchenkov A.S., Khrustalev V.N., Shepel D.V., Nadtochenko V.A., Tskhovrebov A.G. (2021). Femtosecond laser synthesis of nitrogen-doped luminescent carbon dots from acetonitrile. Dye. Pigment..

[11] Stoica G., Castelló Serrano I., Figuerola A., Ugarte I., Pacios R., Palomares E. (2012). Layered double hydroxides as carriers for quantum dots@silica nanospheres. Nanoscale.

[12] Rives V. (2001). Layered Double Hydroxides: Present and Future.

[13] Bian Y., Zhao K., Hu T., Tan C., Liang R., Weng X. (2024). A Se Nanoparticle/MgFe-LDH Composite Nanosheet as a Multifunctional Platform for Osteosarcoma Eradication, Antibacterial and Bone Reconstruction. Adv. Sci..

[14] Wang L., Mao Z., Wu J., Cui X., Wang Y., Yang N., Ge J., Lei H., Han Z., Tang W. (2023). Engineering layered double hydroxide-based sonocatalysts for enhanced sonodynamic-immunotherapy. Nano Today.

[15] Zhang F., He X., Dong K., Yang L., Ma B., Liu Y., Liu Z., Chen B., Zhu R., Cheng L. (2023). Combination therapy with ultrasound and 2D nanomaterials promotes recovery after spinal cord injury via Piezo1 downregulation. J. Nanobiotechnol..

[16] Verdurmen W.P.R., Luginbühl M., Honegger A., Plückthun A. (2015). Efficient cell-specific uptake of binding proteins into the cytoplasm through engineered modular transport systems. J. Control. Release.

[17] Gotman I., Ben-David D., Unger R.E., Böse T., Gutmanas E.Y., Kirkpatrick C.J. (2013). Mesenchymal stem cell proliferation and differentiation on load-bearing trabecular Nitinol scaffolds. Acta Biomater..

[18] Romero-Perez P.S., Dorone Y., Flores E., Sukenik S., Boeynaems S. (2023). When Phased without Water: Biophysics of Cellular Desiccation, from Biomolecules to Condensates. Chem. Rev..

[19] Hozumi K., Otagiri D., Yamada Y., Sasaki A., Fujimori C., Wakai Y., Uchida T., Katagiri F., Kikkawa Y., Nomizu M. (2010). Cell surface receptor-specific scaffold requirements for adhesion to laminin-derived peptide-chitosan membranes. Biomaterials.

[20] Kalawoun H., Obeid M., Ciotonea C., Chaghouri M., Poupin C., Aouad S., Labaki M., Gennequin C., Abi-Aad E., Delattre F. (2023). Review on the contribution of ultrasounds in layered double hydroxides synthesis and in their performances. C. R. Chim..

[21] Silva A.F.D., Duarte J.L.D.S., Meili L. (2021). Different routes for MgFe/LDH synthesis and application to remove pollutants of emerging concern. Sep. Purif. Technol..

[22] da Silva A.F., Duarte J.L.D.S., Georgin J., Franco D.S.P., Selvasembian R., Fernandes D.P., Meili L. (2023). Mechanistic insights of nitrate removal by MgFe/layered double hydroxides prepared by different synthesis pathways. Appl. Surf. Sci. Adv..

[23] Golubev R.A., Rubanik V.V., Kritchenkov I.S., Kritchenkov A.S. (2023). Sorption properties of layered double hydroxides produced by ultrasonic exposure. Front. Mater. Technol..

[24] Golubev R.A., Egorov A.R., Sikaona N.D., Esakova V.E., Semenkova D.I., Liu W., Khrustalev V.N., Kirichuk A.A., Maharramov A.M., Nazarov R.H. (2025). Ultrasonic treatment and sorption properties of Mg^2+^/Fe^3+^ layered double hydroxides. Mendeleev Commun..

[25] Xu H., Zhou H., Hua Y., Chen W., Wu J., Long Z., Zhao L., Wang L., Shen G., Chen Q. (2025). MgFe-LDH engineered biochar self-assembled with Chlorella pyrenoidosa for enhanced CO_2_ capture and microalgal cultivation. Chem. Eng. J..

[26] Salam M.A., Imdadulhaq E.S., Al-Romaizan A.N., Saleh T.S., Mostafa M.M.M. (2023). Ultrasound-Assisted 1,3-Dipolar Cycloadditions Reaction Utilizing Ni-Mg-Fe LDH: A Green and Sustainable Perspective. Catalysts.

[27] Guo Y., Gong Z., Li C., Gao B., Li P., Wang X., Zhang B., Li X. (2020). Efficient removal of uranium (VI) by 3D hierarchical Mg/Fe-LDH supported nanoscale hydroxyapatite: A synthetic experimental and mechanism studies. Chem. Eng. J..

[28] Cui C., Zhang Y., Wladyka M.A., Wang T., Song W., Niu K. (2023). Ultrasound-Assisted Adsorption of Perchlorate Using Calcined Hydrotalcites and the Thermal Stabilization Effect of Recycled Adsorbents on Poly(vinyl chloride). ACS Omega.

[29] Hamzah M.A.A.M., Yusof N., Jaafar J., Ismail A.F., Lau W.J., Salleh W.N.W., Aziz F. (2025). Transforming Palm Oil Fuel Ash into High-Performance Biosorbents: One-Pot Synthesis of Layered Double Hydroxide Composites for Efficient Removal of Hazardous Dyes from Textile Wastewater. Water Air Soil Pollut..

[30] Janani B., Sre V.V., Syed A., Elgorban A.M., Abid I., Wong L.S., Khan S.S. (2025). Engineering defects and lattice disorientation in layered double hydroxides by coupling 2D-Co(OH)_2_ platelets via p-n heterojunction for enhanced photocatalytic degradation chloramphenicol. Colloids Surf. A Physicochem. Eng. Asp..

[31] Scherrer P. (1918). Estimation of the Size and Internal Structure of Colloidal Particles by Means of Röntgen. Nachr. Ges. Wiss. Göttingen Math. Phys. Kl..

[32] Stokes A.R., Wilson A.J.C. (1944). A method of calculating the integral breadths of Debye-Scherrer lines: Generalization to non-cubic crystals. Math. Proc. Camb. Philos. Soc..

[33] Williamson G.K., Hall W.H. (1953). X-ray line broadening from filed aluminium and wolfram. Acta Metall..

[34] Halajnia A., Oustan S., Najafi N., Khataee A.R., Lakzian A. (2012). The adsorption characteristics of nitrate on Mg-Fe and Mg-Al layered double hydroxides in a simulated soil solution. Appl. Clay Sci..

[35] Guwy A.J., Hawkes F.R., Martin S.R., Hawkes D.L., Cunnah P. (2000). A technique for monitoring hydrogen peroxide concentration off-line and on-line. Water Res..

[36] Chabot M., Morales E., Cummings J., Rios N., Giatpaiboon S., Mogul R. (2020). Simple kinetics, assay, and trends for soil microbial catalases. Anal. Biochem..

[37] Johnson J.L., Temple K.L. (1964). Some Variables Affecting the Measurement of “Catalase Activity” in Soil. Soil Sci. Soc. Am. J..

